# *LZTS2* promoter hypermethylation: a potential biomarker for the diagnosis and prognosis of laryngeal squamous cell carcinoma

**DOI:** 10.1186/s12957-018-1349-y

**Published:** 2018-03-02

**Authors:** Zhisen Shen, Lexi Lin, Bing Cao, Chongchang Zhou, Wenjuan Hao, Dong Ye

**Affiliations:** 1 Lihuili Hospital of Ningbo University, No.57, XingNing Road, JiangDong District, Ningbo, China; 2 Children’s Hospital, Zhejiang University School of Medicine, Hangzhou, China; 3 The Medical School of Ningbo University, No.57, XingNing Road, JiangDong District, Ningbo, China

**Keywords:** Laryngeal squamous cell carcinoma, Leucine zipper tumor suppressor 2, DNA methylation, Biomarkers, Tumor suppressor gene

## Abstract

**Background:**

*LZTS2* (leucine zipper tumor suppressor 2), a candidate tumor suppressor gene, suppresses cell growth and plays a vital role in the carcinogenesis and development of tumors. No studies to date have described methylation of the *LZTS2* promoter in human cancers, including LSCC (laryngeal squamous cell carcinoma). Therefore, the aim of this study was to explore the relationship between *LZTS2* promoter methylation and risk of LSCC.

**Methods:**

In our study, *LZTS2* promoter methylation levels in LSCC tumor and adjacent normal tissues from 96 patients were measured using quantitative methylation-specific polymerase chain reaction (qMSP) assays.

**Results:**

The qMSP analyses revealed that *LZTS2* promoter methylation levels in the LSCC tumor samples were significantly higher than those in paired adjacent healthy tissue samples. Furthermore, *LZTS2* methylation levels were elevated in smokers, advanced T classified, and clinically staged patients, as well as in patients with lymph node metastases. In addition, Kaplan-Meier survival curves results showed that overall survival of LSCC patients with hypomethylated LZTS2 promoters was significantly higher than that in patients with hyper-methylated LZTS2 promoters (log-rank test *P* = 0.028). Meanwhile, the area under the receiver operating characteristic curve was 0.920. The diagnostic threshold value for LZTS2 methylation was 11.63% (94.7% sensitivity and 80.4% specificity).

**Conclusions:**

*LZTS2* promoter hypermethylation is associated with risk, progression, and prognosis of LSCC in a cohort of 96 human subjects; *LZTS2* promoter hypermethylation is a candidate diagnostic and prognostic biomarker for LSCC.

## Background

Laryngeal cancer is one of the most common head and neck tumors and has the second highest mortality rates of all respiratory system malignancies [1]. More than 95% of laryngeal carcinomas exhibit laryngeal squamous cell carcinoma (LSCC) pathological features [2]. The pathogenesis of LSCC is complicated and involves genetic, epigenetic, and environmental factors, such as alcohol, tobacco, and asbestos [3]. While recent advancements have been achieved in therapeutic strategies that combine surgery, chemotherapy, and radiotherapy, the 5-year overall survival rate of LSCC remains poor [1, 2].

DNA methylation is a well-studied mechanism driving epigenetic regulation of gene expression [4]. In addition to mutations and deletions, the abnormal methylation of tumor suppressor gene (TSG) promoter is considered to be the third mechanism of inactivation of TSG [4], which mainly happens in the CpG island near the original site of the gene transcription and can lead to the abnormal expression of TSG. Numerous studies have shown that hypermethylation of CpG islands in TSG plays a crucial role in carcinogenesis and progression in various solid and liquid tumors and can be a molecular marker used to identify LSCC [4], such as acute myeloid leukemia [5], bile duct carcinoma [6], and breast cancer [7].

**Fig. 1:**
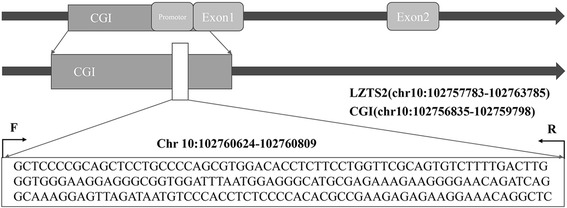
The location of the CpG island and LZTS2 gene promoter. F forward primer, R reverse primer

*LZTS* gene family members share functions related to transcription regulation and controlling the cell cycle [8]. *LZTS2* is located at human 10q24.3 (Fig. 1), which is proximate to 10q23.3, the site of the prototypical tumor suppressor gene *PTEN* [9]. Previous studies have shown that expressions of both regions are frequently downregulated in a variety of tumors, suggesting that other tumor susceptibility genes may exist in these areas in addition to *PTEN* [10]. Mounting evidence supports that *LZTS2* regulates cell growth through protein-protein interactions with β-catenin [11]. β-catenin, one of the subunits of the cadherin protein complex, also performs critical functions in the Wnt signaling pathway. Accumulation of β-catenin in the nucleus plays a vital role in tumorigenesis and progression [12]. *LZTS2* interacts with β-catenin to both repress its transcriptional activity and regulate its subcellular localization and signaling; together, these activities suppress cell growth [11]. In addition, Johnson et al. demonstrated, in *LZTS2* knock-out mouse embryonic fibroblasts, that a lack of *LZTS2* expression promoted cell survival and proliferation [10]. Thus, evidence supports that *LZTS2* is a tumor suppressor gene and that aberrant expression contributes to the genesis and development of some tumors [10].

To date, no reports have investigated methylation of the *LZTS2* promoter in human cancers, including LSCC. Therefore, the aim of the present study was to explore whether correlations exist between *LZTS2* promoter methylation and risk of LSCC.

## Methods

### Patient demographics and tissue sample collection

The study recruited 96 patients who were diagnosed with resectable LSCC tumors. Patients were recruited from the Ear, Nose, Throat, Head, and Neck Surgery Departments of Ningbo Medical Center, Lihuili Hospital. The patients’ median age was 60 years (range 40–86 years; Table 1). The majority of subjects were male (96%). All patients were definitively diagnosed according to criteria established by the World Health Organization [13]. None of the patients received neoadjuvant chemotherapy or radiotherapy, nor did any patient have a family history of LSCC. Patients were followed for up to 58 months. The median follow-up time was 39 months (inter-quartile month range 31–50 months). Fourteen patients were lost to follow-up; twenty-three patients died. Tumor specimens were comprised of 45 well-differentiated cases, 38 moderately differentiated cases, and 13 poorly differentiated cases. Using TMN staging criteria, there were 27 Stage I, 18 Stage II, 11 Stage III, and 40 Stage IV cases. Pathological diagnoses of tumor and paired normal specimens were made in strict accordance with the Union for International Cancer Control classification guidelines (TNM 2002). Specimens were obtained from fresh tissue and then preserved at − 80 °C. Participants signed written informed consent documents. Experimental procedures were reviewed and approved by the Ethics Committee of Ningbo Lihuili Hospital.

**Table 1:** Associations between *LZTS2* promoter methylation and LSCC patient clinicopathological characteristics

Variable	Number	Mean ± SD	*P* value
Gender			
Female	4	20.11 ± 6.39	0.364
Male	92	27.37 ± 15.80	
Age			
≥ 60	49	24.05 ± 14.15	0.995
< 60	47	27.07 ± 17.09	
Smoking behavior			
Yes	78	28.57 ± 15.94	*0.049*
No	18	20.56 ± 12.21	
Histological classification			
Well	45	27.98 ± 15.35	0.590
Moderately/poorly	51	26.25 ± 15.88	
T classification			
T 1 + 2	57	23.26 ± 13.47	*0.003*
T 3 + 4	39	32.62 ± 12.90	
Clinical stage			
Stage I + II	45	22.44 ± 11.64	*0.005*
Stage III + IV	51	31.14 ± 17.48	
Lymph metastasis			
Yes	33	32.85 ± 18.62	*0.019*
No	63	24.03 ± 12.87	

*LZTS2* promoter methylation levels were significantly elevated in advanced stage and advanced T classified patients, in patients who were smokers, as well as cases with lymph node metastasis. Italicized entries indicate statistical significance

### DNA extraction and bisulfite modification

Genomic DNA samples were extracted from tissue specimens using QIAamp DNA Mini Kits (Qiagen, Hilden, Germany) in strict accordance with the manufacturer’s protocols. DNA concentrations and qualities were estimated using a NanoDrop 1000 spectrophotometer (Thermo Fish Scientific Co. Ltd., Wilmington, USA). Eluted DNA was bisulfite-treated using EZ DNA Methylation-Gold Kits following the manufacturer’s protocols (Zymo Research, Irvine, CA, USA).

### Quantification of LZTS2 DNA methylation with quantitative methylation-specific polymerase chain reaction

We measured *LZTS2* gene promoter DNA methylation by applying quantitative methylation-specific polymerase chain reaction (qMSP) technology. The primer sequences were GTTTTTCGTAGTTTTTGTTTTAGCG for the forward primer and AAACCTATTTCCTTCTCTCTTCGAC for the reverse primer (see Fig. 1 for genomic mapping details). Bisulfite-treated DNA from each specimen served as template, and qMSP was performed with FastStart Universal SYBR Green Master Mix (Roche Diagnostics, Mannheim, Germany) and LightCycler 480 real-time PCR amplifier (Roche Diagnostics, Mannheim, Germany) following the operating protocols strictly. The internal controls were designed to ACTB (the forward primer sequences of ACTB were CCTAGAAGCA-TTTGCGGTGG, and the reverse primer sequences were GAGCTACGAGCTGCCTGACG). Bisulfite-treated human genomic DNA served as positive control specimens (Zymo Research, Irvine, CA, USA). Two microliters of bisulfite-treated patient’s DNA was added in 18 μl of PCR reaction mixture containing 10 μl of FastStart Universal SYBR Green Master Mix, 6 μl water, and 1 μl of each forward and reverse primers. The reaction protocol was as follows: 10 min at 95 °C, followed by 50 cycles of 95 °C for 20 s, 60 °C for 40 s, and 72 °C for 30 s. All the procedures were performed in triplicate. The specificity of amplification products was determined with melting curve analyses according to fluorescence data acquired during dissociation steps. Results from the positive control samples were used to construct standard curves for quantification. Each specimen’s percentage of methylated reference (PMR) was calculated by entering cycle threshold (Ct) values for each specimen, internal control, and positive control into the following formula: *PMR* = 2^−[(*CTsample* − *CTinternal*) − (*CTpositive* − *CTinternal*)]^ × 100%.

### Statistical analyses

All statistical tests were performed with SPSS v19.0 (SPSS Inc., Chicago, IL, USA) software. Student’s *t* tests were performed to determine the likelihood that differences observed in *LZTS2* promoter methylation levels between malignant and paired normal tissues occurred due to chance alone. Further paired-samples *t* test was performed on *LZTS2* promoter methylation rates taking into consideration the following clinicopathological characteristics: gender, age, smoking status, histological classification, T classification, presence of lymph node metastases, and clinical stage. Overall survival curves were evaluated by Kaplan-Meier analyses, and statistical differences between curves were evaluated using log-rank tests. Univariate and multivariate Cox proportional hazard models were used to test the prognostic value of *LZTS2* methylation for LSCC patients. A two-tailed *P* < 0.05 was considered to be significant. Figures were illustrated using SPSS v19.0 and GraphPad Prism 7 software (GraphPad, San Diego, CA, USA).

## Results

**Fig. 2:**
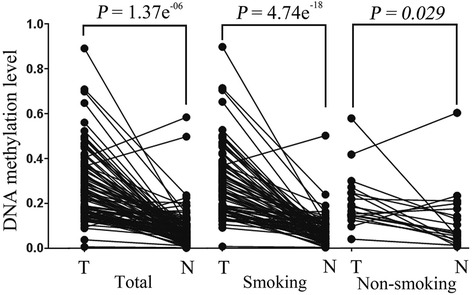
Comparison of *LZTS2* methylation levels between LSCC malignant tissues and paired adjacent normal tissues: *LZTS2* promoter methylation levels were significantly higher in LSCC tissues compared to normal tissues (*n* = 96, *P* = 1.37e^−06^). Stratification analyses according to smoking status indicated the difference of *LZTS2* promoter methylation levels between LSCC tissues and normal tissues was more significant in smokers (*n* = 78, *P* = 4.74e^−18^) versus non-smokers (*n* = 18, *P* = 0.029). T tumor specimen, N normal adjacent specimen

*LZTS2* promoter methylation levels were evaluated in 96 LSCC specimens and paired adjacent normal tissues using qMSP technologies. The present data revealed that *LZTS2* promoter methylation levels were significantly higher in LSCC cancer tissues when compared to paired adjacent normal tissues (Fig. 2, *P* = 1.37e^−06^). Given that smokers are at risk of developing LSCC [14], the cohort was stratified according to smoking status. Subgroup analyses revealed that the observed *LZTS2* hypermethylation patterns occurring in LSCC (when compared to paired normal tissues) were more clearly evident in smokers than non-smokers (Fig. 2, smoking *P* = 4.74e^−18^; non-smokers *P* = 0.029). Next, associations between clinicopathological characteristics (e.g., age, gender, smoking behavior, histological classification, T classification, clinical stage, and lymph node metastases) and *LZTS2* promoter methylation rates were further explored (Table 1). As expected, the subgroup analyses confirmed that *LZTS2* promoter methylation levels of LSCC in smokers were significantly elevated, relative to non-smokers (*P* = 0.049). Additionally, T classification, clinic stage, and presence of lymph metastases all served as significant explanatory variables. The PMR in advanced clinical stages (stages III + IV) and T classifications (T III + IV) were significantly greater than specimens exhibiting earlier clinical stages (stages I + II; *P* = 0.005) and lower T classifications (T I + II; *P* = 0.003). In contrast to patients without lymph node metastases, methylation levels of patients with lymph node metastases were significantly elevated (*P* = 0.019). The mean of PMR in male subjects, subjects over 60 years old, and subjects with well-differentiated histologies were numerically higher than female subjects, subjects under 60 years old, and subjects with moderately and poorly differentiated histologies, respectively; however, these differences were not statistically significant (Table 1).

**Fig. 3:**
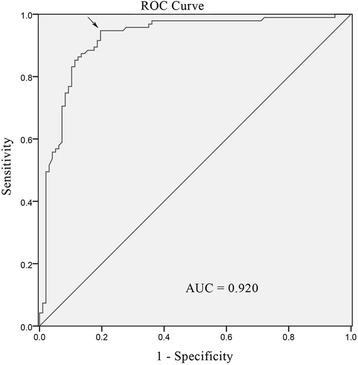
The ROC analysis of the curve. The cut-off point was defined as the maximum Youden index, which was demarcated by the arrow in the figure

To evaluate the potential diagnostic value of *LZTS2* methylation, the receiver operating characteristic (ROC) curve was plotted. This technique served to identify a diagnostic threshold value for *LZTS2* promoter methylation. The area under the ROC curve (AUC) was 0.920 (Fig. 3). The diagnostic threshold value (cut-off value) for *LZTS2* methylation was 11.63% (94.7% sensitivity and 80.4% specificity). The values over the cut-off were defined as positive diagnostic indicators, while those below the cut-off were considered negative indicators. The false positive and false negative rates were 18.8 and 5.2%, respectively. The positive predictive value was 83.5% and negative predictive value was 94.0%. The diagnostic accordance rate was 88.0%.

**Fig. 4:**
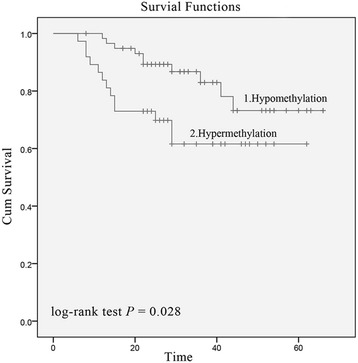
Kaplan-Meier curves of overall survival in *LZTS2* promoter hypomethylated and hypermethylated LSCC patients. Log-rank test results indicated that LSCC patients with *LZTS2* hypermethylated promoters (*n* = 37) had significantly worse overall survival rates than those who had *LZTS2* hypomethylated promoters (*n* = 59) (*P* = 0.028)

To evaluate whether an association existed between patient overall survival and *LZTS2* methylation, survival analyses were performed on two subcohorts that were split at the average LSCC PMR value (average value: 0.2706). Fifty-nine patients were classified into the hypomethylation group and 37 were assigned to the hypermethylation group. Using Kaplan-Meier analyses, overall survival of LSCC patients with hypomethylated *LZTS2* promoters was significantly higher than that of patients with hypermethylated *LZTS2* promoters (Fig. 4, log-rank test *P* = 0.028). Univariate Cox proportional hazards analysis also revealed an obviously increased risk of death for LSCC patients with hypermethylated *LZTS2* (HR = 44.366; 95% CI = 4.586–429.237; *P* = 0.001). Subsequently, we performed a multivariate Cox proportional hazard analysis by adjusting for smoking behavior, histological differentiation, clinical stage, and lymphatic metastasis. The results confirmed that *LZTS2* promoter hypermethylation could be an independent factor to predict a poorer overall survival of LSCC patients (HR = 6.671; 95% CI = 2.087–21.324; *P* = 0.001, Table 2).

**Table 2:** Multivariate Cox proportional hazards analysis of the 96 LSCC patients

Characteristics	Number	*P* value	HR	95% CI
Smoking behavior				
No (Ref)	18	–	1	–
Yes	78	0.888	1.084	0.356–3.295
Histological classification				
Well (Ref)	45	–	1	–
Moderately/poorly	51	0.995	1.003	0.421–2.392
Clinical stage				
Stage I + II (Ref)	45	–	1	–
Stage III + IV	51	0.822	1.181	0.278–5.012
Lymph metastasis				
No (Ref)	63	–	1	–
Yes	33	0.127	2.657	0.757-9.327
*LZTS2* methylation	96	0.011	20.184	1.979-205.847

*Ref* reference category, *HR* hazard ratio, *CI* confidence interval

## Discussion

For LSCC patients, early-stage disease often goes undetected because symptoms are mild and non-specific. Consequently, roughly 40% of newly diagnosed patients present with stage III or IV disease [15]. Currently, the prevailing initial therapy for LSCC is total laryngectomy combined with postoperative radiotherapy. Given that the larynx plays an indispensable role in human communication and that the operative treatment damages laryngeal structure and function, current treatment paradigms greatly reduce patients’ quality of life [13]. Patients for whom disease is identified in T1 or T2 stages have an 80–90% cure rate; for advanced LSCC, cure rates drop to about 60% [2]. Therefore, novel methods are needed to allow for earlier identification and possible prevention of LSCC, while cancers remain curable and laryngeal function can be best preserved.

Abnormal TSG promoter methylation is an early and frequent event occurring in LSCC tumorigenesis [4]. Accumulating evidence indicates that hypermethylated gene promoters exist in LSCC tissue, as well as in the serum and saliva of LSCC patients [4]. It stands to reason that hypermethylation of a panel of TSGs could serve LSCC patients as sensitive diagnostic and prognostic biomarkers, or as markers following the natural history of curative therapies [4].

Here, a novel TSG was evaluated. *LZTS2* is located at human 10q24.3, is expressed in most normal tissues [8], suppresses cell growth by interacting with β-catenin [11], and plays a vital role in the carcinogenesis and development of tumors [10]. To date, research has focused on the *LZTS2* TSG functions; however, the relationship between *LZTS2* promoter methylation events and tumorigenesis was largely unstudied. This study investigated associations between *LZTS2* promoter methylation and risk of LSCC, as well as *LZTS2* clinical diagnostic value. Previous studies have shown that *LZTS2* cDNA overexpression suppressed cell growth and reduced colony formation rates of several cancer cell lines, including HEK-293T, AT6.2, LNCaP, PC3, TRSUPr1, Rat-1, and U2OS [8]. Additionally, *LZTS2* protein has low expression levels in human prostate cancer cells [10]. The present results revealed that *LZTS2* promoter methylation levels in LSCC tissues were significantly higher than in non-cancerous paired control specimens. Thus, the data suggested that *LZTS2* promoter hypermethylation decreased *LZTS2* protein expression, which may have regulated LSCC carcinogenic mechanisms and increased risk of developing the disease.

Next, clinicopathologic parameters were identified that served as explanatory variables contributing to *LZTS2* methylation levels. Previous studies demonstrated that, in addition to lung cancer, cigarette smoking is a risk factor for upper aero-digestive tract cancers including laryngocarcinomas, hypopharyngeal carcinomas, and esophageal cancers [14]. Additionally, smoking gives rise to extensive genome-wide DNA changes especially DNA methylation, which play important roles in tumorigenesis [16]. Therefore, the current study specifically investigated a relationship between *LZTS2* promoter methylation and cigarette smoking. *LZTS2* promoter methylation levels in LSCC specimens from smokers were significantly elevated, relative to non-smokers (*P* = 0.049). Thus, smoking behavior may have elevated *LZTS2* promoter methylation levels and thus increased risk of LSCC in those patients. Additionally, *LZTS2* promoter methylation levels were significantly increased in patients with lymph node metastases, advanced clinical stages, and advanced T classifications. It is well-understood that T classification and lymph node metastases are crucial to the prognosis of LSCC patients [17]. The present results suggested that *LZTS2* promoter methylation also contributed to LSCC progression and ultimate prognosis. Furthermore, previous studies have reported that abnormal TSG methylation was associated with poor survival outcomes in several tumor types [5–7]. Survival analyses presented here indicated that patients with *LZTS2* promoter hypermethylation have worsened outcomes compared to those with hypomethylation in the same promoter regions (Fig. 3), which was consistent with the results of our univariate Cox proportional hazards analysis. Furthermore, the multivariate Cox proportional hazard analysis we performed confirmed that *LZTS2* methylation was an independent adverse factor for LSCC outcomes. These findings suggested that *LZTS2* promoter hypermethylation could be a potential biomarker for prognosis of LSCC.

The advent of tumor-specific biomarkers has made major strides in advancing clinic diagnostic practices in a variety of tumors, including CEA and CA19-9 for colorectal cancer [18], AFP for hepatocellular carcinoma [19], and PSA for prostate cancer [20]. However, no biomarker currently exists to assist in clinical prognostication of laryngeal cancer. Here, the AUC of the curve of ROC was 0.920; a large AUC is indicative of a high diagnostic value. The sensitivity, specificity, and diagnostic accordance rates were 94.7, 80.4, and 88.0%, respectively. These data provided strong evidence that *LZTS2* promoter hypermethylation could be clinically applicable for use in early identification of LSCC.

## Conclusion

*LZTS2* promoter hypermethylation was associated with LSCC risk, progression, and prognosis, with the strongest associations observed in the subgroup of patients who smoked. Taken together, *LZTS2* promoter hypermethylation has the potential to become an LSCC diagnostic and prognostic biomarker.

## Abbreviations

AUC: Area under the ROC curve
CI: Confidence interval
CT: Cycle threshold
HR: Hazard ratio
LSCC: Laryngeal squamous cell carcinoma
PMR: Percentage of methylated reference
qMSP: Quantitative methylation-specific polymerase chain reaction
ROC: Receiver operating characteristic
TSG: Tumor suppressor gene
